# Independent Assessment of the Children’s Hepatic Tumors International Collaboration Risk Stratification for Hepatoblastoma and the Association of Tumor Histological Characteristics With Prognosis

**DOI:** 10.1001/jamanetworkopen.2021.48013

**Published:** 2022-02-11

**Authors:** Shengmei Zhou, Jemily Malvar, Yueh-Yun Chi, James Stein, Larry Wang, Yuri Genyk, Richard Sposto, Leo Mascarenhas

**Affiliations:** 1 Department of Pathology and Laboratory Medicine, Children’s Hospital Los Angeles, Los Angeles, California; 2 Keck School of Medicine, University of Southern California, Los Angeles, California; 3 Cancer and Blood Disease Institute, Division of Hematology and Oncology, Department of Pediatrics, Children’s Hospital Los Angeles, Los Angeles, California; 4 Department of Surgery, Children’s Hospital Los Angeles, Los Angeles, California

## Abstract

**Importance:**

Hepatoblastoma is the most common pediatric liver malignant neoplasm, and accurate risk stratification is essential for guiding treatment.

**Objective:**

To validate the Children’s Hepatic Tumors International Collaboration–Hepatoblastoma Stratification (CHIC-HS) in an independent cohort of patients with hepatoblastoma and evaluate the association of pretreatment hepatoblastoma histological subtype with prognosis.

**Design, Setting, and Participants:**

This is a single-institution retrospective cohort study of 96 pediatric patients with hepatoblastoma diagnosed and treated between June 1, 2000, and December 31, 2016, with recent therapy and independent of the CHIC-HS discovery cohort. Each patient was assigned a risk group according to CHIC-HS. The histological characteristics of each tumor were assessed based on the International Pediatric Liver Tumor Consensus Classification. Data were analyzed from May 2018 to May 2019.

**Main Outcomes And Measures:**

The main outcomes were event-free survival (EFS) and overall survival (OS). Cox regression analysis was used to examine the associations of patient characteristics and tumor histological characteristics with survival.

**Results:**

A total of 96 patients (median [range] age, 1.9 [0.4-18] years; 36 [38%] girls and 60 [63%] boys) were assessed, including 15 with very low risk, 28 with low risk, 23 with intermediate risk, and 30 with high risk, according to CHIC-HS criteria. There were a total of 13 cancer-related deaths; median (range) follow-up was 3.5 (0.1-17.8) years for those alive at the last follow-up. The estimated 5-year OS rates were 100% in the very low-risk group, 94.7% (95% CI, 68.1%-99.2%) in the low-risk group, 89.2% (95% CI, 63.1%-97.2%) in the intermediate-risk group, and 57.9% (95% CI, 34.6%-75.5%) in the high-risk group. In a multivariable analysis, we confirmed that CHIC-HS significantly estimated EFS (high-risk group vs very low- and low-risk groups: hazard ratio [HR], 45.59; 95% CI, 9.39-209.5; *P* < .001) and OS (high-risk group vs very low- and low-risk groups: HR, 21.95; 95% CI, 2.76-174.29; *P* < .001). In the subcohort of 84 patients for whom pretreatment tumor histological data were available, tumor epithelial histological subtypes were found to be significantly associated with both EFS and OS. Patients in the CHIC-HS high-risk group and with embryonal-only histological subtype had the highest risk of relapse or disease progression (high-risk: HR, 42.62; 95% CI, 9.91-203.9; embryonal: HR, 3.28; 95% CI, 1.21-8.9) and death (high-risk: HR, 18.78; 95% CI, 2.31-152.84; embryonal: HR, 7.12; 95% CI, 1.51-33.52).

**Conclusions and Relevance:**

This cohort study found that CHIC-HS performed as expected in an independent cohort that was more recently treated. Incorporation of pretreatment tumor histological data into CHIC-HS may provide additional prognostic value.

## Key Points

**Question:**

Is Children’s Hepatic Tumor International Collaboration–Hepatoblastoma Stratification (CHIC-HS) risk group prognostic of outcomes and are tumor histological characteristics associated with risk stratification?

**Findings:**

In this cohort of 96 pediatric patients with hepatoblastoma from a single institution who were independent of the CHIC-HS discovery cohort and treated with more recent therapy, CHIC-HS group was significantly associated with both event-free survival and overall survival. Data on the contribution of hepatoblastoma histologic subtype to risk stratification were also generated.

**Meaning:**

These findings validate the CHIC-HS and suggest that incorporating pretreatment tumor histological characteristics into CHIC-HS may provide additional prognostic value.

## Teaser

This cohort study assesses the Children’s Hepatic Tumors International Collaboration-Hepatoblastoma Stratification in an independent cohort of patients with hepatoblastoma and evaluates the association of pretreatment tumor histological characteristics with prognosis.

## Introduction

Hepatoblastoma is the most common pediatric liver malignant neoplasm, typically occurring in patients between ages 6 months and 3 years.^1^ The overall survival for patients with hepatoblastoma has improved dramatically in the last 2 decades, with approximately 80% of patients surviving long term.^2^ However, the prognosis for patients with high-risk hepatoblastoma remains poor, and most survivors of hepatoblastoma experience adverse effects of chemotherapy, such as permanent hearing loss, nephrotoxic effects, and bone marrow suppression.^3^ Refining risk stratification for hepatoblastoma has the potential for guiding clinicians to make informed decisions on therapy: surgical treatment followed by observation for patients with very low risk, less toxic chemotherapy for patients with low and intermediate risk, further intensification of chemotherapy for patients with high risk, and early consideration for liver transplantation when indicated.

Many clinicopathological features, such as pretreatment extent of disease (PRETEXT) stage, distant metastasis at diagnosis, serum α-fetoprotein (AFP) level at diagnosis, age at diagnosis, tumor histological characteristics, and genetic markers, have been reported to be prognostic for survival.^4,5,6,7,8^ Over the last 2 decades, 4 different risk stratification systems for hepatoblastoma were used by 4 major pediatric liver cancer cooperative clinical trial groups: International Childhood Liver Tumor Strategy Group (SIOPEL), Children’s Oncology Group (COG), German Society of Pediatric Oncology and Hematology (GPOH), and Japanese Study Group for Pediatric Liver Tumors (JPLT).^9,10^ Owing to different risk stratifications, it is difficult to compare the results of clinical trials from these 4 groups. Furthermore, given the rarity of this malignant neoplasm, collaboration among the 4 clinical trial groups is required to advance clinical outcomes in children with hepatoblastoma. To overcome this, leaders from these 4 groups came together to form a consensus group called the Children’s Hepatic Tumors International Collaboration (CHIC). Data from 8 prospective clinical trials conducted by these 4 groups were pooled to form the CHIC database, which included 1605 patients with hepatoblastoma treated between 1988 and 2008.^9^ Recently, CHIC developed a new consensus international risk stratification system for hepatoblastoma called Children’s Hepatic Tumors International Collaboration–Hepatoblastoma Stratification (CHIC-HS).^10^ CHIC-HS categorizes patients into 4 risk groups based on the following prognostic factors: PRETEXT stage, PRETEXT annotation factors, presence or absence of distant metastases, patient age at diagnosis, AFP level at diagnosis, and tumor resectability at diagnosis. Tumor histological characteristics were conspicuously not included, owing to sparse data. CHIC-HS is being implemented in an international prospective collaborative treatment trial, Paediatric Hepatic International Tumor Trial (PHITT) (NCT03017326), that includes patients with hepatoblastoma. However, to date, CHIC-HS has not been independently validated. The aim of this study was to assess the performance of CHIC-HS in an independent cohort and to investigate whether pretreatment tumor histological data might add value to CHIC-HS.

## Methods

This cohort study was approved by the institutional review board of the Children’s Hospital Los Angeles (CHLA). Informed consent was waived by the CHLA review board because the study was a retrospective review and it was not possible to contact all patients.

The surgical pathology archives of CHLA were searched for all patients diagnosed with hepatoblastoma, transitional liver cell tumor, or hepatocellular neoplasm not otherwise specified (HCN-NOS) from June 1, 2000, to December 31, 2016. A total of 197 samples from 122 patients were found, 100 of whom were diagnosed and treated at our institution. Demographic data, clinical features, AFP levels at diagnosis, PRETEXT stage and annotation factors, tumor resectability, disease status, and vital status were collected by retrospective review of electronic medical records, radiological imaging, and pathology reports, and 98 of 100 patients had all the necessary information to be accurately classified by CHIC-HS. Two patients were excluded because they were included in the CHIC-HS discovery cohort. The CHIC risk stratification tree^10^ was used to determine each patient’s risk group in our analytic cohort of 96 patients.

An immunohistochemistry panel of glypican 3, β-catenin and Sal-like protein 4 were performed in most samples. All hematoxylin and eosin and immunohistochemistry slides from tumor biopsy, resection, or liver explant were rereviewed by ^2^ pathologists (S.Z. and L.W.). Based on the International Pediatric Liver Tumor Consensus Classification,^11^ each tumor was first assigned broadly to the histological type: epithelial vs mixed epithelial-mesenchymal variant, and further to 1 of the epithelial histological subtypes based on epithelial components: fetal, embryonal, fetal + embryonal (regardless of relative proportions of each component), HCN-NOS, and others, including 6 with small cell undifferentiated (SCU) histological characteristics, 1 with cholangioblastic histological characteristics, and 1 with macrotrabecular pattern. Representative examples of epithelial components are provided in the eFigure in the Supplement. Samples with discordance were adjudicated by consensus.

### Statistical Analysis

The χ^2^ test was used to examine differences in the distribution of patient characteristics and CHIC-HS risk strata. The primary end points were event-free survival (EFS) and overall survival (OS). EFS was defined as the interval between the date of diagnosis and the date of first relapse of disease, disease progression, or death, whichever occurred first. OS was defined as the interval between the date of diagnosis and the date of death from any cause. Patients without an event were censored at the time of last follow-up. Cox regression analysis was used to examine the associations of patient and tumor epithelial histological features with EFS and OS. Demographic- and tumor-related traits used in the CHIC-HS risk stratification, such as age at diagnosis, AFP levels, and PRETEXT stage, were excluded from the univariable and multivariable analyses since the association between these factors and the CHIC-HS risk strata are highly correlated. Factors examined in univariable models included patient sex, transplant, CHIC-HS risk strata, and tumor epithelial subtype histological features. Significant factors from the univariable analysis (ie, those with *P* < .05) were considered in the multivariable analysis. The final model was determined by a backward stepwise selection when all factors remaining achieved a likelihood ratio test *P* < .05. Owing to small sample size, the CHIC-HS very low- and low-risk groups were combined in the analysis. Tumors with SCU histological features were incorporated into the others group owing to small sample size. Well-differentiated and crowded fetal histological features were not analyzed separately. Twelve patients lacked pretreatment tumor histological data. Therefore, the association of tumor epithelial histological features among all patients and among 84 patients with pretreatment tumor histological data were examined separately. The Kaplan-Meier method^12^ was used to assess EFS and OS. The survival estimates included 95% CIs, which were calculated using the log-log transformation and Greenwood formula. Unless otherwise stated, all analyses were performed using 2-sided tests with significance set at *P* < .05 and completed using Stata statistical software version 2015 (StataCorp). Data were analyzed from May 2018 to May 2019.

## Results

### Clinicopathologic Characteristics

A total of 96 patients (median [range] age, 1.9 [0.4-18] years; 36 [38%] girls and 60 [63%] boys) formed the analytic cohort. There were 15 patients in the very low-risk group, 28 patients in low-risk group, 23 patients in the intermediate-risk group, and 30 patients in the high-risk group, according to CHIC-HS criteria. The associations among CHIC-HS risk group and various clinicopathologic features are listed in Table 1. Most patients (64 patients [67%]) were younger than 3 years, and 8 patients (8%) were older than 8 years. Five patients had AFP levels of 100 ng/mL or lower (after ruling out hook effect by serial dilution; to convert to micrograms per liter, multiply by 1) at diagnosis, including 3 patients in the very low-risk group and 2 patients in the high-risk group. Two patients had foci of SCU histological characteristics in the tumor, 2 patients had fetal + embryonal subtype, and 1 patient had fetal subtype. Most patients had PRETEXT stage III or IV tumors (71 patients [74%]). Most of the tumors had epithelial histological characteristics (82 patients [85%]). The predominant epithelial subtypes were fetal + embryonal (43 patients [45%]), followed by embryonal (20 patients [21%]). Of 10 patients with HCN-NOS histological characteristics, 9 patients were in the CHIC-HS high-risk group: 4 patients experienced a relapse or disease progression and 5 underwent liver transplantation. However, none of the patients with HCN-NOS had died by the time of last follow-up. A total of 28 patients (29%) underwent liver transplantation, mainly in the intermediate-risk (13 patients) and high-risk (9 patients) groups. After a median (range) follow-up of 3.5 (0.1-17.8) years among those alive at last follow-up, there were a total of 26 EFS events and 13 deaths (all disease related). The overall 5-year EFS was 67.3% (95% CI, 54.9%-77.1%), and the overall 5-year OS was 81.8% (95% CI, 70.4%-89.2%) (Figure, A). CHIC-HS risk group was significantly associated with age at diagnosis, AFP level, PRETEXT stage, PRETEXT annotation factors, metastasis, epithelial histological characteristics, liver transplant, events, and death (Table 1).

**Table 1.** CHIC-HS Risk Group and Clinicopathologic Characteristics

Characteristics	CHIC-HS risk group, No. (%)				*P* value
	Very low (15)	Low (28)	Inter (23)	High (30)	
Sex					
Girls	9 (60)	11 (39)	8 (35)	8 (27)	.18
Boys	6 (40)	17 (61)	15 (65)	22 (73)	
Age at diagnosis, y^a^					
<3	10 (67)	25 (89)	17 (74)	12 (40)	<.001
3-8	5 (33)	3 (11)	6 (26)	10 (33)	
≥8	0	0	0	8 (27)	
Serum AFP, ng/mL^a^					
≤100	3 (20)	0	0	2 (7)	.007
101-1000	3 (20)	1 (4)	5 (22)	1 (3)	
>1000	9 (60)	27 (96)	18 (78)	27 (90)	
PRETEXT stage^a^					
I	10 (67)	1 (4)	1 (4)	1 (3)	<.001
II	5 (33)	2 (7)	2 (9)	3 (10)	
III	0	25 (89)	14 (61)	12 (40)	
IV	0	0	6 (26)	14 (47)	
VPEFR^a^					
Negative	15 (100)	28 (100)	5 (22)	12 (40)	<.001
Positive	0	0	18 (78)	18 (60)	
Tumor metastasis^a^					
Absent	15 (100)	28 (100)	23 (100)	11 (37)	<.001
Present	0	0	0	19 (63)	
Histological characteristics					
Epithelial	13 (87)	24 (86)	19 (83)	26 (87)	.98
Mixed	2 (13)	4 (14)	4 (17)	4 (13)	
Epithelial subtype					
Fetal	4 (27)	4 (14)	5 (22)	2 (7)	.002
Embryonal	2 (13)	5 (18)	6 (26)	7 (23)	
Fetal + embryonal	5 (33)	18 (64)	10 (43)	10 (33)	
HCN-NOS	0	0	1 (4)	9 (30)	
Others	4 (27)	1 (4)	1 (4)	2 (7)	
Transplant					
No	15 (100)	22 (79)	10 (43)	21 (70)	.002
Yes	0	6 (21)	13 (57)	9 (30)	
Events					
No	15 (100)	26 (93)	20 (87)	9 (30)	<.001
Yes	0	2 (7)	3 (13)	21 (70)	
Died					
No	15 (100)	27 (96)	21 (91)	20 (67)	.002
Yes	0	1 (4)	2 (9)	10 (33)	

Abbreviations: AFP, α-fetoprotein; CHIC-HS, Children’s Hepatic Tumors International Collaboration risk stratification for hepatoblastoma; HCN-NOS, hepatocellular neoplasm not otherwise specified; PRETEXT, pretreatment extent of disease; VPEFR, PRETEXT annotation factors.

SI conversion factor: To convert AFP to micrograms per liter, multiply by 1.

^a^ These variables were incorporated in CHIC-HS.

**Figure.**
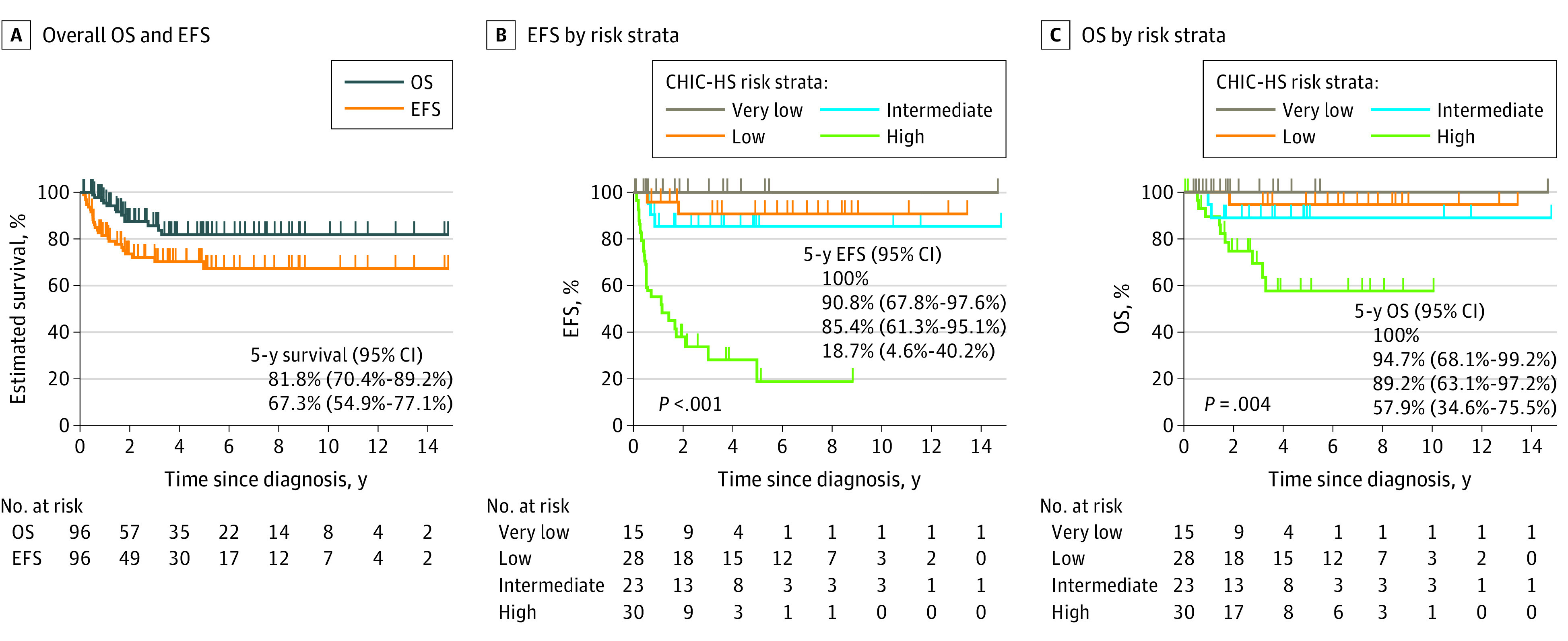
Overall Survival (OS) and Event-Free Survival (EFS) Overall and by Children’s Hepatic Tumors International Collaboration Risk Stratification for Hepatoblastoma (CHIC-HS) Group Crosses indicate censored patients.

### Performance of CHIC-HS

The results of the univariable analysis of EFS and OS in the full cohort of 96 patients are provided in Table 2, and multivariable analysis results are in Table 3. Univariable analysis found that CHIC-HS was significantly associated with both EFS and OS (Table 2). Compared with patients in the very low- and low-risk groups, patients in the high-risk group were at significantly higher risk of death (hazard ratio [HR], 15.17; 95% CI, 1.94-118.70). Compared with the very low- and low-risk groups, the difference in risk for patients in the intermediate-risk group was not statistically significant (HR, 4.18; 95% CI, 0.38-46.18). The difference among groups was significant (*P* = .001). Multivariable analysis found significant differences in EFS among groups (*P* < .001). Compared with patients in the very low-and low-risk groups, there was a higher risk of disease relapse or progression in the high-risk group (HR, 45.59; (95% CI, 9.39-209.5); the difference in risk was not statistically significant for the intermediate-risk group (HR, 3.26; 95% CI, 0.54-19.83). The risk of death also varied significantly among groups (*P* < .001). Risk of death was also higher in the high-risk group (HR, 21.95; 95% CI, 2.76-174.29), but the difference for the intermediate-risk group was not statistically significant (HR, 3.72; 95% CI, 0.33-41.53) (Table 3). We repeated the multivariable analysis on the subcohort of 84 patients with pretreatment tumor histological data available. There were 24 EFS events and 12 deaths in this subcohort, and we found similar results to the main analysis (Table 4). Both CHIC-HS and epithelial histological characteristics were independent risk factors for both EFS and OS. The 5-year EFS rates were 100% for the very low-risk group, 90.8% (95% CI, 67.8%-97.6%) for the low-risk group, 85.4% (95% CI, 61.3%-95.1%) for the intermediate-risk group, and 18.7% (95% CI, 4.6%-40.2%) for the high-risk group (Figure, B), and the 5-year OS rates were 100% for the very low-risk group, 94.7% (95% CI, 68.1%-99.2%) for the low-risk group, 89.2% (95% CI, 63.1%-97.2%) for the intermediate risk group, and 57.9% (95% CI, 34.6%-75.5%) for the high-risk group (Figure, C).

**Table 2.** Univariate Cox Regression Analysis of Survival in the Full Cohort

Patient or tumor characteristics	Event-free survival		Overall survival	
	HR (95% CI)	*P* value	HR (95% CI)	*P* value
CHIC-HS group				
Very low or low	1 [Reference]	<.001	1 [Reference]	.001
Intermediate	3.06 (0.51-18.38)		4.18 (0.38-46.18)	
High	23.72 (5.53-101.78)		15.17 (1.94-118.70)	
Histological characteristics (epithelial vs mixed)	0.67 (0.23-1.97)	.57	0.28 (0.08-1.06)	.10
Epithelial subtype				
Fetal	1 [Reference]	.36	1 [Reference]	.02
Embryonal	3.01 (0.79-11.42)		8.22 (1.01-67.02)	
Fetal + embryonal	1.22 (0.32-4.62)		1.63 (0.17-15.77)	
Others	2.03 (0.41-10.05)		3.71 (0.34-40.90)	
HCN-NOS	1.99 (0.45-8.91)		NA	
Epithelial regrouped				
Fetal with or without Embryonal	1 [Reference]	.23	1 [Reference]	.007
Embryonal	2.61 (1.05-6.51)		5.84 (1.71-19.97)	
Other	1.76 (0.49-6.32)		2.23 (0.48-14.42)	
HCN-NOS	1.73 (0.55-5.44)		NA	
Male sex (vs female)	2.10 (0.84-5.23)	.09	1.93 (0.53-7.01)	.32
Transplant (vs no transplant)	0.8 (0.34-1.90)	.61	0.37 (0.08-1.66)	.16

Abbreviations: HR, hazard ratio; CHIC-HS, Children’s Hepatic Tumors International Collaboration risk stratification for hepatoblastoma; HCN-NOS, hepatocellular neoplasm, not otherwise specified; NA, not applicable.

**Table 3.** Multivariable Cox Regression Analysis of Survival in the Full Cohort

Patient or tumor characteristics	Event-free survival		Overall survival	
	HR (95% CI)	*P* value	HR (95% CI)^a^	*P* value
CHIC-HS group				
Very low or low	1 [Reference]	<.001	1 [Reference]	<.001
Intermediate	3.26 (0.54-19.83)		3.72 (0.33-41.53)	
High	45.59 (9.39-209.5)		21.95 (2.76-174.29)	
Epithelial subtype				
Fetal with or without Embryonal	1 [Reference]	.004	1 [Reference]	<.001
Embryonal	2.66 (1.02-6.94)		4.41 (1.28-15.19)	
Other	2.14 (0.58-7.85)		2.17 (0.39-12.00)	
HCN-NOS	0.28 (0.08-0.94)		NA	

Abbreviations: HR, hazard ratio; CHIC-HS, Children’s Hepatic Tumors International Collaboration risk stratification for hepatoblastoma; HCN-NOS, hepatocellular neoplasm, not otherwise specified; NA, not applicable.

^a^ With the small ratio of the number of events to the number of parameters, the HR estimates may be biased and should be interpreted with caution.

**Table 4.** Multivariable Cox Regression Analysis of Survival in the Subcohort Patients With Pretreatment Tumor Histological Data (n = 84)

Patient or tumor characteristics	Event-free survival		Overall survival	
	HR (95% CI)	*P* value	HR (95% CI)^a^	*P* value
CHIC-HS group				
Very low or low	1 [Reference]	<.001	1 [Reference]	<.001
Intermediate	2.69 (0.36-20.23)		1.99 (0.12-32.83)	
High	42.62 (8.91-203.90)		18.78 (2.31-152.84)	
Epithelial subtype				
Fetal with or without Embryonal	1 [Reference]	.001	1 [Reference]	<.001
Embryonal	3.28 (1.21-8.90)		7.12 (1.51-33.52)	
Other	2.14 (0.44-10.49)		2.66 (0.23-30.38)	
HCN-NOS	0.30 (0.08-1.07)		NA	

Abbreviations: CHIC-HS, Children’s Hepatic Tumors International Collaboration risk stratification for hepatoblastoma; HR, hazard ratio; HCN-NOS, hepatocellular neoplasm, not otherwise specified; NA, not applicable.

^a^ With the small ratio of the number of events to the number of parameters, the HR estimates may be biased and should be interpreted with caution.

### Prognostic Role of Tumor Epithelial Histological Subtype

We first evaluated the associations of 3 different histological classifications with EFS and OS in all patients by univariate Cox regression analysis (Table 2). The histological classification (epithelial vs mixed epithelial-mesenchymal variant) did not show statistical significance in estimating EFS and OS. The epithelial subtype classification, using fetal as the reference, was significantly associated with OS (embryonal: HR, 8.22; 95% CI, 1.01-67.02; fetal + embryonal: HR 1.63; 95% CI, 0.17-15.77; others: HR, 3.71; 95% CI, 0.34-40.90) (*P* = .02), but there were no associations with EFS. When regrouped by epithelial subtype histological characteristics, histological subtype remained significantly associated with OS (embryonal: HR, 5.84; 95% CI, 1.71-19.97; other: HR, 2.23; 95% CI, 0.48-14.42) (*P* = .007) but not EFS (Table 2). In the multivariable analysis, we assessed epithelial subtype classification and found that epithelial subtype was independently significantly associated with EFS (embryonal: HR, 2.66; 95% CI, 1.02-6.94; other: HR, 2.14; 95% CI, 0.58-7.85) (*P* = .004) and OS (embryonal: HR, 4.41; 95% CI, 1.28-15.19; other: 2.17; 95% CI, 0.39-12.00) (*P* < .001) (Table 3). Tumors with HCN-NOS histological characteristics had a protective association with risk of disease relapse or progression compared with tumors with some fetal histological characteristics (HR, 0.28; 95% CI, 0.08-0.94). Next, we assessed the prognostic role of epithelial histological characteristics in EFS and OS by including only patients who had pretreatment tumor histological data. Multivariable analysis found that epithelial subtypes were still independently associated with both EFS (embryonal: HR, 3.28; 95% CI, 1.21-8.90; other: HR, 2.14; 95% CI, 0.44-10.49; HCN-NOS: HR, 0.30; 95% CI, 0.08-1.07) (*P* = .001) and OS (embryonal: HR, 7.12; 95% CI, 1.51-33.52; other: HR, 2.66; 95% CI, 0.23-30.38) (*P* < .001) (Table 4). We also found that integration of pretreatment tumor epithelial histological data into CHIC-HS provided additional prognostic value. The patients in the CHIC-HS high-risk group and with only embryonal histological characteristics had the highest risk of relapse or progression (9 of 9 patients) or death (8 of 9 patients), while the patients in CHIC-HS very low- and low-risk groups and with some fetal histological characteristics had the lowest risk of relapse or progression (1 of 26 patients) or death (0 of 26 patients).

## Discussion

This cohort study is the first independent validation of CHIC-HS and the largest study to investigate the prognostic role of tumor histological characteristics in hepatoblastoma, to our knowledge. Accurate risk stratification of patients with hepatoblastoma could improve patient care by optimizing treatment, facilitating clinical trial design, fostering international collaboration and the development of new therapeutic approaches, and improving communication among physicians. Over the last decade, individual clinical trial groups have attempted to define the relative importance of a variety of suspected prognostic factors.^5,7,13^ Poor prognostic factors identified individually in 4 clinical trials included PRETEXT stage IV, distant metastasis, AFP less than 100 ng/mL, and SCU histological characteristics.^7^ Recently, CHIC developed the most refined risk stratification system for hepatoblastoma by taking into account most known prognostic factors.

CHIC-HS performed well in our cohort. In the multivariable analysis, we found that CHIC-HS was the main prognostic factor for outcome and independently significantly estimated both EFS and OS. Notably, CHIC-HS maintained its value in a more recently treated group of patients with contemporary therapies. Our treatment approach during the study period was upfront surgical resection of PRETEXT stage I or II primary tumors followed by either observation for patients with pure fetal histological characteristics, or postoperative adjuvant chemotherapy for patients with other histological characteristics. For patients who had unresectable primary tumors at presentation, a biopsy was performed when feasible, followed by neoadjuvant chemotherapy, delayed tumor resection, including liver transplantation if indicated, and postoperative adjuvant chemotherapy. In our cohort, all patients in the very low-risk group had low PRETEXT stage, and 26 of 30 patients in the high-risk group had high PRETEXT stage. The 5-year OS estimate (81.8%) in our cohort was similar to the survival rate (81.9%) reported from the Surveillance, Epidemiology and End Results database.^14^ We also found that the estimated mortality closely matched observed mortality across the CHIC-HS risk groups.

Additionally, we found that integration of pretreatment epithelial histological data into CHIC-HS provided additional prognostic value. Previous clinical trials have identified that some histological types are associated with prognosis. For example, patients with pure well-differentiated fetal histological characteristics are likely to have an excellent outcome with complete primary tumor excision^13^; while patients with SCU histological characteristics are more likely to require more aggressive therapy and have a poorer prognosis than patients with other histological characteristics.^15,16^ Both well-differentiated fetal and SCU histological characteristics were incorporated in COG risk stratification and therapeutic protocols.^2^ Patients diagnosed with stage I pure well-differentiated fetal hepatoblastomas in COG AHEP0731 were treated with surgical resection alone.^3^ However, tumor histological characteristics were not incorporated in this initial CHIC-HS owing to lack of complete histological data. Historically, COG recommended biopsy or upfront primary resection before any chemotherapy was administered, when feasible. Therefore, pretreatment histological data were available for patients enrolled in COG trials. In contrast, the other 3 major clinical trial groups (SIOPEL, GPOH, and JPLT) recommended neoadjuvant chemotherapy prior to surgical resection; therefore, most patients only had postchemotherapy histological data.^11^ Of 96 patients in our cohort, 84 patients had pretreatment tumor histological data, which was assessed based on the International Consensus Histology Classification. Our data showed that pretreatment epithelial histological characteristics were independently significantly associated with EFS and OS. The patients in CHIC-HS high-risk group and with embryonal-only histological characteristics had the highest risk of progression, relapse, or death, while the patients in CHIC-HS very low- and low-risk groups and with some fetal histological characteristics had the lowest risk of adverse outcomes.

In this study, we had 2 patients with pure well-differentiated fetal histological characteristics. Both patients were classified in the very low-risk group and were cured with surgical treatment alone. Six patients had tumors with focal SCU histological characteristics (<1% to 80% of tumor), which were all INI-1 retained; 5 of these patients were previously reported.^17^ Four of these 5 patients were alive without evidence of disease. The sixth patient with focal SCU histological characteristics was also alive at the last follow-up. Although tumors with focal SCU histological characteristics were not separately analyzed owing to low case numbers, our data suggested that INI-1–retained SCU histological characteristics might not be an unfavorable prognostic factor. Of 10 patients with HCN-NOS histological characteristics, 9 patients were in the CHIC-HS high-risk group and 1 patient was in the intermediate-risk group. The contrarian excellent outcomes noted in patients with HCN-NOS are likely to be related to surgical intervention, including liver transplantation.^18^ All patients with HCN-NOS histological characteristics had complete resections of the primary tumor, and 5 of 10 patients underwent liver transplantation to achieve complete resection status. Ideally, any risk stratification should be independent of the results of any therapeutic modality. However, it is well known that complete tumor resection is a prerequisite for cure of hepatoblastoma and that any strategy leading to an increased resection rate will result in improved outcomes for patients with hepatoblastoma.^5^

Very low serum AFP (<100 ng/mL) is included in CHIC-HS as a poor prognostic factor. An association between SCU histological characteristics and AFP levels that are low or with reference range for age has been suggested.^5,15^ In this study, 5 patients had very low serum AFP at diagnosis. Of these, 2 patients had focal SCU histological characteristics, 2 patients had fetal + embryonal histological characteristics, and 1 patient had fetal histological characteristics. Only 1 of these patients died owing to complications following liver transplant.^17^ Our limited data suggest that very low AFP was not associated with either tumor histological characteristics or prognosis. Malignant rhabdoid tumors (loss of INI-1) are reported to develop in the liver and are often misdiagnosed as SCU hepatoblastomas.^19,20^ It is perhaps reasonable to speculate that some of malignant rhabdoid tumors may have been misclassified as low-AFP hepatoblastomas in the past.

### Limitations

This study has several limitations, including its retrospective nature, a relatively small sample size, and shorter follow-up time for patients diagnosed after 2014. Given the small number of patients and events in some epithelial histological subgroups, estimates from the multivariate models are prone to bias, and the corresponding 95% CIs can be imprecise. Therefore, these results should be interpreted with caution and thus, the contribution of histological characteristics to risk stratification should be validated independently and prospectively.

## Conclusions

The findings of this cohort study provide evidence to support the use of CHIC-HS in PHITT and advance previous knowledge on the prognostic role of tumor histological characteristics in hepatoblastoma. It is planned that PHITT will evaluate the role of pretreatment tumor histological characteristics within the context of the international multigroup collaborative clinical trial and elucidate more precisely the prognostic role of histological characteristics in hepatoblastoma, paving the way forward for the inclusion of pretreatment diagnostic histological characteristics into risk stratification for patients newly diagnosed with hepatoblastoma.

## Notes

Supplement. **eFigure.** Representative Examples of 4 Major Components of Hepatoblastoma
