# Supplementary material for: Independent Assessment of the Children’s Hepatic Tumors International Collaboration Risk Stratification for Hepatoblastoma and the Association of Tumor Histological Characteristics With Prognosis
Source: JAMA Netw Open. 2022 Feb 11;5(2):e2148013. doi: 10.1001/jamanetworkopen.2021.48013 (PMC8837914; doi:10.1001/jamanetworkopen.2021.48013)
Supplement: Supplement. — eFigure. Representative Examples of 4 Major Components of Hepatoblastoma [file jamanetwopen-e2148013-s001.pdf]

Supplemental Online Content

Zhou S, Malvar J, Chi YY, et al. Independent assessment of the Children's Hepatic Tumors International Collaboration risk stratification for hepatoblastoma and the association of tumor histological characteristics with prognosis. *JAMA Netw Open*. 2022;5(2):e2148013. doi:10.1001/jamanetworkopen.2021.48013

eFigure. Representative Examples of 4 Major Components of Hepatoblastoma

This supplemental material has been provided by the authors to give readers additional information about their work.

© 2022 Zhou S et al. *JAMA Network Open.*

eFigure. Representative examples of 4 major components of hepatoblastoma.

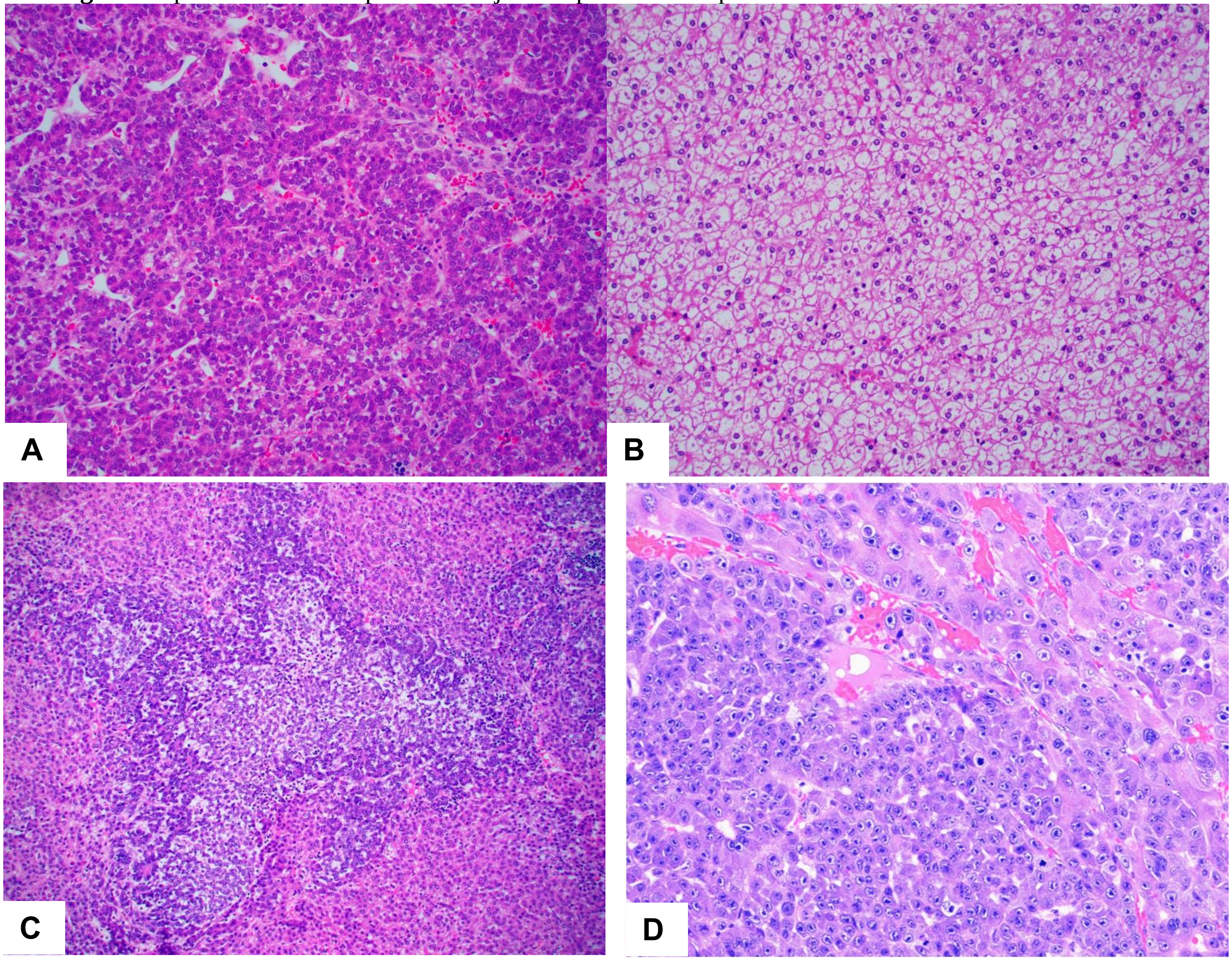

© 2022 Zhou S et al. *JAMA Network Open.*

A. Embryonal component: Tumor is composed of medium sized round to oval hyperchromatic vesicular nuclei,small nucleoli, scanty to moderate amounts of eosinophilic cytoplasm, numerous mitoses and arranged mainly in acini and trabeculae. B. Fetal component: The tumor is composed of sheets ofsmall sized polygonal cells with centrally located small vesicular nuclei, inconspicuous nucleoli and abundant clear to eosinophilic cytoplasm without mitoses. C. Small cell undifferentiated component: The tumor is composed of nests of small to medium sized, loosely cohesive cells with open chromatin, inconspicuous nucleoli and scant pale to eosinophilic cytoplasm, consistentwith SCU components (middle of the photo) surrounded by embryonal components (dark colored) which were further surrounded by fetal components (pink colored). D. Hepatocellular neoplasm, NOS (HCN-NOS): The tumor is composed of sheets of medium to large polygonal cells with large nuclei, prominent nucleoli, abundant eosinophilic granular cytoplasm and many mitoses. (A-D, Hematoxylin-eosin stain; Original magnification: 200 x for A, B and D, 100 x for C)

© 2022 Zhou S et al. *JAMA Network Open.*
